# Effect of *NOTCH3* EGFr Group, Sex, and Cardiovascular Risk Factors on CADASIL Clinical and Neuroimaging Outcomes

**DOI:** 10.1161/STROKEAHA.122.039325

**Published:** 2022-07-13

**Authors:** Remco J. Hack, Minne N. Cerfontaine, Gido Gravesteijn, Stephan Tap, Anne Hafkemeijer, Jeroen van der Grond, Marie-Noëlle Witjes-Ané, Frank Baas, Julie W. Rutten, Saskia A.J. Lesnik Oberstein

**Affiliations:** Department of Clinical Genetics (R.J.H., M.N.C., G.G., S.T., F.B., J.W.R., S.A.J.L.O.), Leiden University Medical Center, the Netherlands.; Department of Geriatrics and Psychiatrics (M.N.W.-A.), Leiden University Medical Center, the Netherlands.; Department of Radiology (A.H., J.v.d.G.), Leiden University Medical Center, the Netherlands.; Institute of Psychology (A.H.), Leiden University, the Netherlands.; Leiden Institute for Brain and Cognition (A.H.), Leiden University, the Netherlands.

**Keywords:** CADASIL, hypertension, mutation, neuroimaging, risk factors, sex, white matter

## Abstract

A retrospective study has shown that EGFr (epidermal growth factor–like repeat) group in the *NOTCH3* gene is an important cerebral autosomal dominant arteriopathy with subcortical infarcts and leukoencephalopathy (CADASIL) disease modifier of age at first stroke and white matter hyperintensity (WMH) volume. No study has yet assessed the effect of other known CADASIL modifiers, that is, cardiovascular risk factors and sex, in the context of *NOTCH3* EGFr group. In this study, we determined the relative disease-modifying effects of *NOTCH3* EGFr group, sex and cardiovascular risk factor on disease severity in the first genotype-driven, large prospective CADASIL cohort study, using a comprehensive battery of CADASIL clinical outcomes and neuroimaging markers.

**Methods:**

Patients with CADASIL participated in a single-center, prospective cohort study (DiViNAS [Disease Variability in NOTCH3 Associated Small Vessel Disease]) between 2017 and 2020. The study protocol included a clinical assessment, neuropsychological test battery and brain magnetic resonance imaging on a single research day. Multivariable linear, logistic and Cox regression models were used to cross-sectionally assess the effect of CADASIL modifiers on clinical severity (stroke, disability, processing speed) and neuroimaging markers (WMH volume, peak width of skeletonized mean diffusivity, lacune volume, brain volume, cerebral microbleed count).

**Results:**

Two hundred patients with CADASIL participated, of which 103 harbored a *NOTCH3* EGFr 1–6 variant and 97 an EGFr 7–34 variant. *NOTCH3* EGFr 1–6 group was the most important modifier of age at first stroke (hazard ratio, 2.45 [95% CI, 1.39–4.31]; *P*=0.002), lacune volume (odds ratio, 4.31 [95% CI, 2.31–8.04]; *P*=4.0×10^-6^), WMH volume (B=0.81 [95% CI, 0.60–1.02]; *P*=1.1×10^-12^), and peak width of skeletonized mean diffusivity (B=0.65 [95% CI, 0.44–0.87]; *P*=1.6×10^-8^). EGFr 1–6 patients had a significantly higher WMH volume in the anterior temporal lobes and superior frontal gyri and a higher burden of enlarged perivascular spaces. After *NOTCH3* EGFr group, male sex and hypertension were the next most important modifiers of clinical outcomes and neuroimaging markers.

**Conclusions:**

*NOTCH3* EGFr group is the most important CADASIL disease modifier not only for age at first stroke and WMH volume but also strikingly so for a whole battery of clinically relevant disease measures such as lacune volume and peak width of skeletonized mean diffusivity. *NOTCH3* EGFr group is followed in importance by sex, hypertension, diabetes, and smoking.

Cerebral autosomal dominant arteriopathy with subcortical infarcts and leukoencephalopathy (CADASIL) is a hereditary small vessel disease (SVD) caused by pathogenic cysteine altering variants located in the *NOTCH3* gene (*NOTCH3*^cys^).^1,2^ SVD severity in CADASIL is highly variable, ranging from first stroke in the third decade to symptom-free survival up to the eighth decade.^3,4^ The variability in CADASIL disease severity is poorly understood, but *NOTCH3*^cys^ variant location in one of the 34 EGFr (epidermal growth factor-like repeat) domains of the NOTCH3 ectodomain has been shown to be the most important modifier in CADASIL in retrospective studies: *NOTCH3*^cys^ variants located in one of the first 6 EGFr domains (EGFr 1–6) have been found to be associated with a higher risk of stroke^5-7^ and higher white matter hyperintensity (WMH) volume^5^ than *NOTCH3*^cys^ variants located in one of EGFr domains 7 to 34 (EGFr 7–34). However, much is still to be learned about the differences in SVD phenotype between EGFr 1–6 and EGFr 7–34 patients with CADASIL, as no study has yet compared the burden of all relevant SVD imaging markers, clinical symptoms and cognitive function between the *NOTCH3*^cys^ EGFr groups. Moreover, all previous studies investigating *NOTCH3^cys^* EGFr group were retrospective in nature, which could have led to substantial bias.

Next to *NOTCH3*^cys^ EGFr group, cardiovascular risk factors (CVRF)^8-17^ and sex^10,12,13,15^ have been shown to modulate CADASIL disease severity. However, the relative impact of the various modifiers is unknown, as EGFr group has never been included in studies addressing CADASIL disease modifiers.

Here, we performed a prospective, genotype-driven, single-center cohort study of 200 uniformly characterized patients with CADASIL called DiViNAS (Disease Variability in NOTCH3 Associated Small Vessel Disease), with an equal representation of patients with EGFr 1–6 and EGFr 7–34 variants. We investigated for the first time differences in disease severity between these EGFr groups using extensive clinical and neuroimaging phenotyping, including clinical data of the affected parent of each participant. We also included less well studied CADASIL imaging markers, such as enlarged perivascular spaces and diffusion tensor imaging (DTI) metrics, and determined the relative disease-modifying effects of EGFr group, CVRF, and sex on clinical and neuroimaging SVD outcomes.

## Methods

### Data Availability

The data that support the findings of this study are available from the corresponding authors upon reasonable request.

### DiViNAS Cohort

All patients and presymptomatic family members in the Dutch CADASIL registry with a genetically confirmed *NOTCH3*^cys^ variant were contacted and asked to participate in the DiViNAS prospective, genotype-driven cohort study. All individuals in the DiViNAS cohort were 20 years or older and were included between November 2017 and December 2020. Each participant completed the full study protocol at the research site (Leiden University Medical Center), with clinical assessment, neuropsychological test battery and brain magnetic resonance imaging performed on a single day at the same study site by the same researchers for all participants.

The study was approved by the medical ethics committee of the Leiden University Medical Center (P18.164 and P17.170). All participants gave written informed consent. This manuscript follows the STROBE (Strengthening the Reporting of Observational studies in Epidemiology) reporting guideline.^18^

### Assessment of Clinical Symptoms and Cardiovascular Risk factors

The following clinical outcomes were compared between EGFr 1–6 and EGFr 7–34 patients: age at onset and presence of stroke, transient ischemic attack, dementia, depression, apathy, migraine with/without aura, encephalopathy, walking disabilities, seizures, cognitive test scores, and disability assessed with modified Rankin Scale (mRS). Details about the definition of the clinical symptoms and CVRF can be found in the Supplemental Methods.^19^

Also, the risk of stroke and death was assessed in the affected parent of each participant by determining the age at first stroke and age at death on clinical record, family history, and the Personal Records Database of the Dutch government, respectively. The affected parent of each participant was determined based on genetic testing (n=81) or on clinical and family history (n=46). The affected parent could not be determined in 13 participants.

Laboratory evaluation included hemoglobin A1C and a nonfasting lipid panel (LDL-C [low-density lipoprotein cholesterol], HDL-C [high-density lipoprotein cholesterol], and triglycerides).

### Cognitive Examination

The cognitive examination battery included Montreal Cognitive Assessment, Trail Making Test A and B, Stroop Test, Rey Auditory Verbal Learning Test, Verbal Fluency animal and professions, Wechsler Adult Intelligence Test IV Block design, Coding, and Digit Span. An established composite score of processing speed was calculated by averaging t scores of Trail Making Test A and B.^20^ Details about the calculation of cognitive test scores can be found in the Supplemental Methods.^21,22^

### Neuroimaging Acquisition Parameters

Brain magnetic resonance imagings were all performed on the same 3 Tesla MR system (Philips Achieva TX, Philips Medical Systems, Best, the Netherlands) and evaluated according to consensus criteria.^23^ The brain magnetic resonance imaging protocol included the following sequences: 3-dimensional T1-weighted images, T2-weighted images, fluid-attenuated inversion recovery, susceptibility-weighted images, and diffusion-weighted imaging sequences. Acquisition parameters are presented in the Supplemental Methods.

### Quantification of Conventional SVD Imaging Markers and DTI Metrics

The following conventional SVD markers were evaluated according to consensus criteria^23^: normalized WMH volume (nWMHv), normalized lacune volume (nLV), cerebral microbleed (CMB) count, brain parenchymal fraction (BPF), and burden of enlarged perivascular spaces (ePVS). BPF was defined as the ratio of brain parenchymal volume to the intracranial volume expressed as percentage. WMH and lacune volume were normalized to the intracranial volume (normalized volume=[unnormalized volume/intracranial volume]×100). ePVS were evaluated in 4 specific regions: the global white matter, basal ganglia, subinsular region and anterior temporal lobes (ATL), according to a 4-grade semiquantitative scale.^24^ As WMH in the ATL and superior frontal gyri (SFG) have been shown to be frequently present in CADASIL and lead to a higher brain volume due to increased water content,^25,26^ nWMHv was also assessed in these regions separately and used as a covariate in the analyses with BPF as outcome.

The following DTI metrics were quantified on preprocessed diffusion-weighted images: peak width of the skeletonized mean diffusivity (PSMD), fractional anisotropy, mean, axial, and radial diffusivity. PSMD, which is a proven robust imaging marker for SVD,^27^ is the difference between the 95th and 5th percentiles of the voxel-based mean diffusivity values within the skeleton.

Details about the quantification of the conventional SVD imaging markers and DTI metrics can be found in the Supplemental Methods.^24,26-28^

### Statistics

Normally distributed continuous variables were summarized using mean±SD and compared between 2 groups using unpaired *t* tests. Non-normally distributed continuous variables were summarized using median with interquartile range. Binary categorical variables were compared between 2 groups using Fisher exact Tests.

In all analyses, continuous variables were standardized by dividing their value by their SD. The following variables were transformed to obtain a normal distribution or a linear relationship between dependent and independent variables in multiple linear regression: total nWMHv (square root), nWMHv in ATL and SFG (ln transformed), nLV (cube root), CMB count (log_10_[x+1]), fractional anisotropy (squared), other diffusion metrics (mean diffusivity, axial diffusivity, radial diffusivity; natural log), Trail Making Test t scores (squared), Montreal Cognitive Assessment score (reflect and cube root). CMB count (total and per location), and nLV were divided in quartiles.

Multiple linear and logistic regression models were used to compare clinical symptoms, cognitive scores and SVD neuroimaging markers between EGFr 1–6 and EGFr 7–34 patients, corrected for age (*P*_age_). In time-to-event analysis, log-rank test was used to compare age at onset of clinical symptoms between the 2 groups (*P*_LR_).

Multiple linear, logistic, and Cox regression models were used to assess the effect of EGFr group, sex, and CVRF on 8 different SVD outcomes: age at first stroke, processing speed, mRS score, nWMHv, nLV, BPF, CMB count, and PSMD. To assess the association of hemoglobin A1C or nonfasting lipid levels (LDL-C, HDL-C and triglycerides) with disease severity, diabetes and hypercholesterolemia were excluded as covariates, respectively. Bonferroni correction was used to correct for multiple testing of 8 different SVD outcomes (*P* value/8).

All statistical analyses were performed in R (version 4.1.0). Two-sided *P*<0.05 was considered statistically significant.

## Results

### DiViNAS Cohort

A total of 200 patients with CADASIL from the Dutch CADASIL registry were recruited in the DiViNAS cohort, of which 97 patients harbored a *NOTCH3*^cys^ variant located in one of EGFr domains 1–6 and 103 patients in one of EGFr domains 7–34 (Table S1).

EGFr 1–6 patients were significantly younger than EGFr 7–34 patients: mean age 48.9±12.3 years (range, 26–75) versus mean age 55.6±11.3 years (range, 29–81; *P*=8.6×10^-5^; Table 1). EGFr 1–6 patients had a significantly higher educational level than EGFr 7–34 patients (*P*=0.012). Although there were no significant differences in CVRF, EGFr 7–34 patients more often had diabetes type 1 or 2 (*P*=0.08), hypertension (*P*=0.15), and hypercholesterolemia (*P*=0.14).

**Table.**
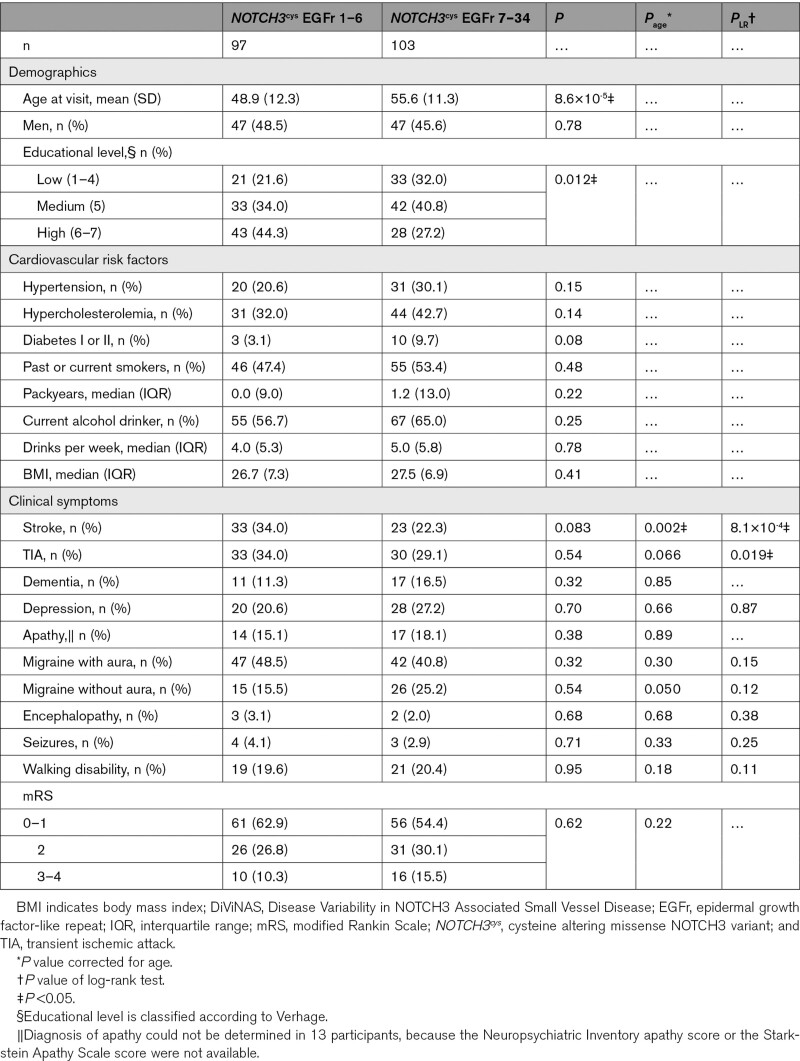
Demographics, Cardiovascular Risk Factor Profiles and Clinical Symptoms of the DiViNAS Cohort

### Differences in Clinical, Neuropsychological and Neuroimaging Outcomes Between EGFr 1–6 Patients and EGFr 7–34 Patients

#### Clinical and Neuropsychological Outcomes

**Figure 1.**
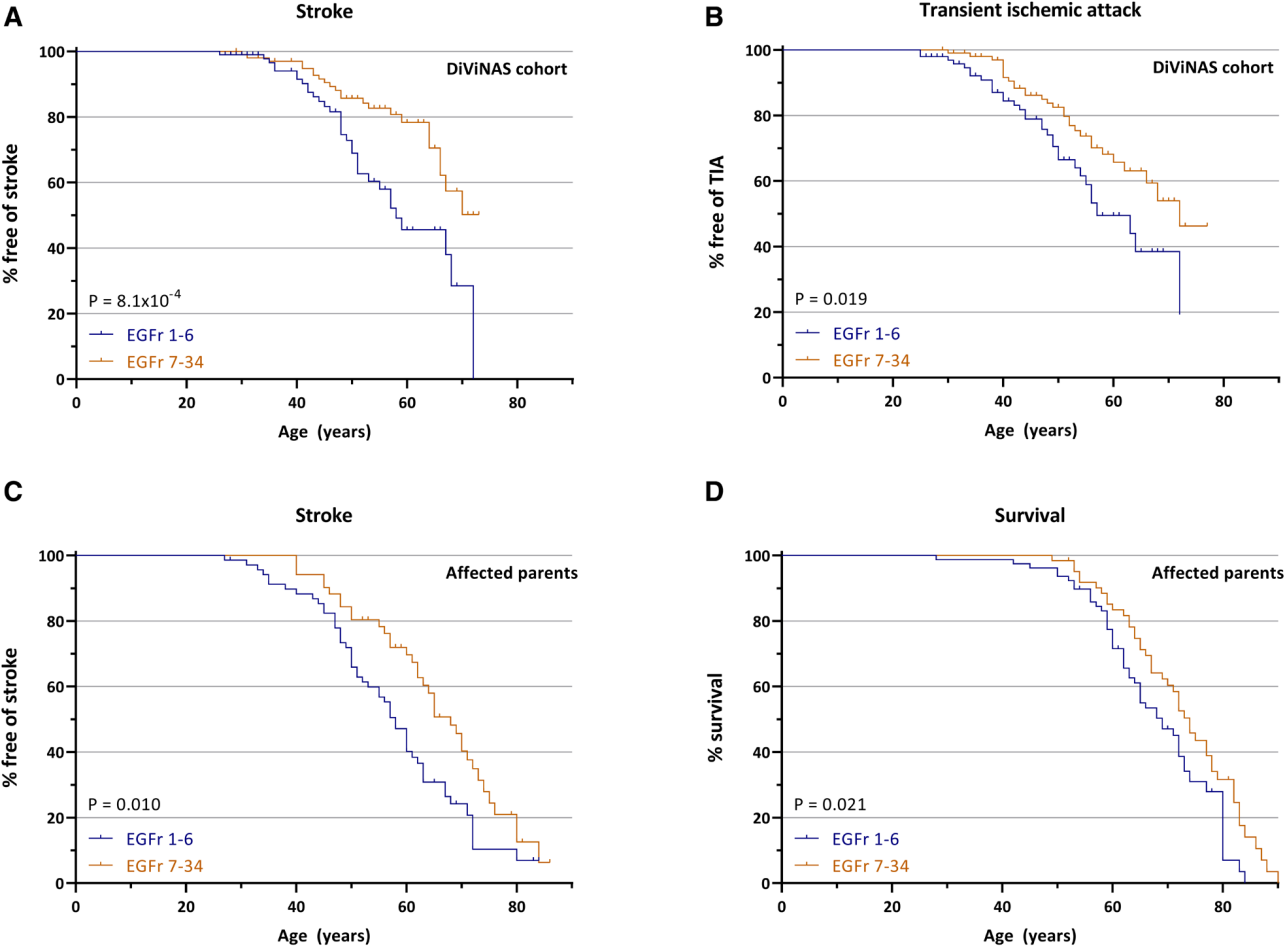
**Cysteine altering NOTCH3 variants (*NOTCH3*^cys^) located in epidermal growth factor-like repeat (EGFr) domains 1–6 are associated with an increased risk of stroke and lower survival compared to EGFr 7–34 *NOTCH3*^cys^ variants.** Kaplan-Meier plots showing that EGFr 1–6 patients have a significantly earlier onset of stroke (median 58 vs >73 y; *P*_LR_=8.1×10^-4^; **A**) and transient ischemic attack (TIA; median 57 vs 72 y; *P*_LR_=0.019; **B**), than EGFr 7–34 patients. Affected parents of EGFr 1–6 patients have an earlier onset of stroke (median 58 vs 68 y; *P*_LR_=0.010; **C**) and a lower life expectancy (median 69 y vs 74 y; *P*_LR_=0.021; **D**), than affected parents of EGFr 7–34 patients. The affected parent of 13 patients with a *NOTCH3*^cys^ EGFr 7–34 variant could not be discerned as neither parent had obvious cerebral autosomal dominant arteriopathy with subcortical infarcts and leukoencephalopathy (CADASIL)–associated signs or symptoms. DiViNAS indicates Disease Variability in NOTCH3 Associated Small Vessel Disease.

EGFr 1–6 patients had a significantly earlier onset of stroke than EGFr 7–34 patients (median 58 versus >73; *P*_LR_=8.1×10^-4^) and transient ischemic attack (median 57 versus 72 years; *P*_LR_=0.019; Figure 1A and 1B). Affected parents of EGFr 1–6 patients also had a significantly earlier onset of stroke than those of EGFr 7–34 patients (median 58 versus 68 years; *P*_LR_=0.010) and a lower life expectancy (median 69 years versus 74 years; *P*_LR_=0.021; Figure 1C and 1D). After adjustment for age, there was no significant difference in mRS score (odds ratio [OR], 1.42 [95% CI, 0.81–2.48]; *P*=0.22) or cognitive test scores (all *P*>0.16) between EGFr 1– 6 and EGFr 7–34 patients (Table S2). There was also no significant difference in presence and age at onset of any of the following: migraine with aura, dementia, depression, seizures, walking disability, and CADASIL encephalopathy.

#### Neuroimaging Outcome Measures

**Figure 2.**
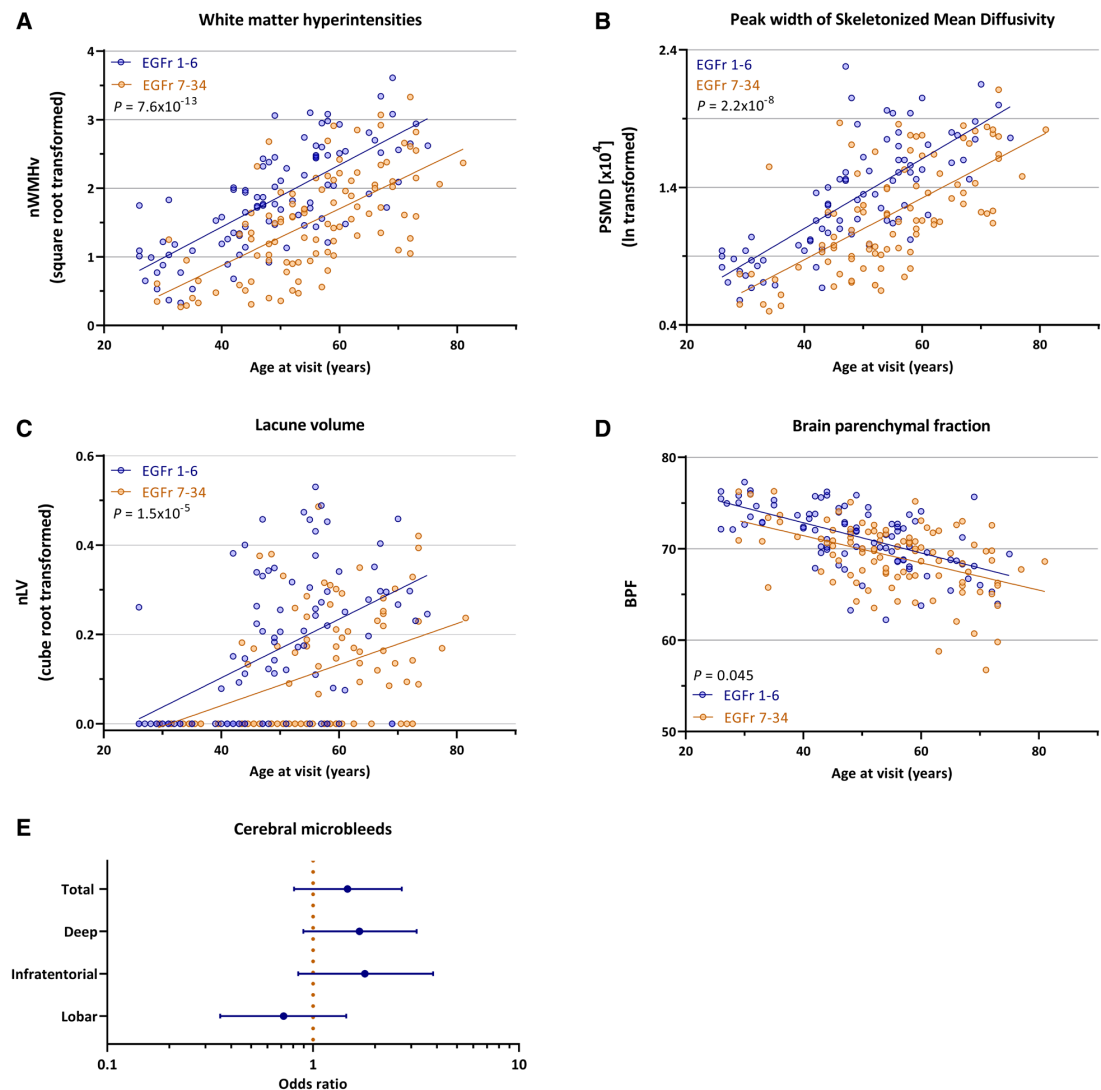
**Cysteine altering NOTCH3 variants (*NOTCH3*^cys^) located in EGFr (epidermal growth factor-like repeat) domains 1–6 are associated with a higher white matter hyperintensity volume, peak width of skeletonized mean diffusivity, lacune volume, and brain parenchymal fraction compared to EGFr 7–34 *NOTCH3*^cys^ variants.** Scatterplots showing that EGFr 1–6 patients have a significantly higher normalized white matter hyperintensity volume (nWMHv; B=0.80 [95% CI, 0.60–1.01]; *P*=7.6×10^-13^; **A**), peak width of skeletonized mean diffusivity (PSMD; B=0.65 [95% CI, 0.43–0.86]; *P*=2.2×10^-8^; **B**), normalized lacune volume (nLV; odds ratio [OR], 3.74 [95% CI, 2.07–6.92]; *P*=1.8×10^-5^; **C**), and brain parenchymal fraction (BPF; B=0.24 [95% CI, 0.01–0.48]; *P*=0.045; **D**) than EGFr 7–34 patients. (**E**) There were no significant difference in cerebral microbleed count (total and per region). The odds ratio is shown for EGFr 1–6 patients compared to EGFr 7–34 patients (reference group: OR=1).

**Figure 3.**
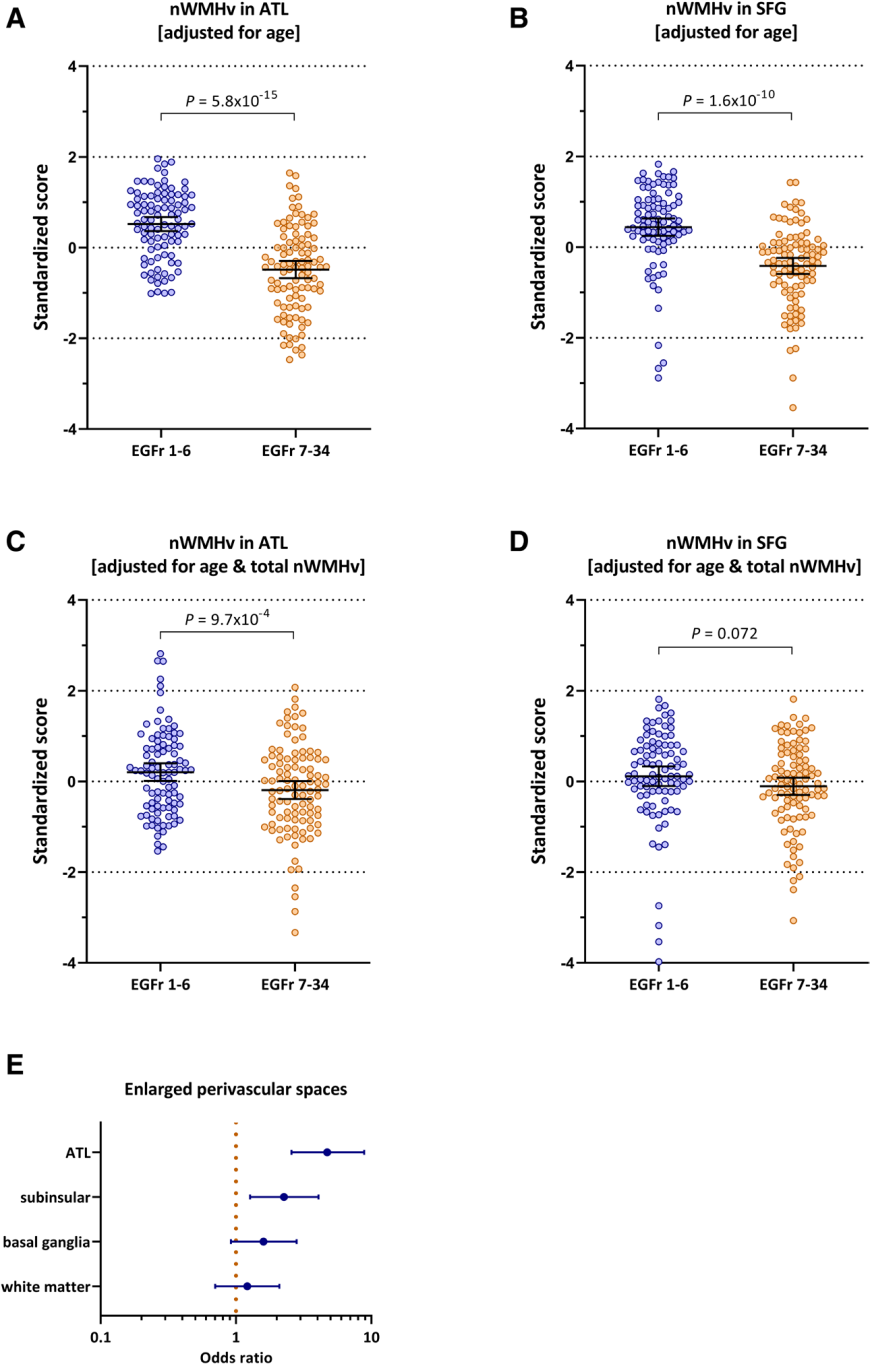
**Cysteine altering NOTCH3 variants (*NOTCH3*^cys^) located in epidermal growth factor-like repeat (EGFr) domains 1–6 are associated with a higher burden of white matter hyperintensity volume and enlarged perivascular spaces in cerebral autosomal dominant arteriopathy with subcortical infarcts and leukoencephalopathy (CADASIL)–specific regions, that is, anterior temporal lobes and superior frontal gyri, than EGFr 7–34 *NOTCH3*^cys^ variants. A** and **B**, EGFr 1–6 patients have a significantly higher normalized white matter hyperintensity volume (nWMHv) in the anterior temporal lobes (ATL; B=1.00 [95% CI, 0.77–1.23]; *P*=5.8×10^-15^) and superior frontal gyri (SFG; B=0.83 [95% CI, 0.59–1.07]; *P*=1.6×10^-10^) than EGFr 7–34 patients. **C** and **D**, After adjustment for total nWMHv, this remained significant for nWMHv in the ATL (B=0.26 [95% CI, 0.11–0.42]; *P*=9.7×10^-4^; **C**) and was borderline significant for SFG (B=0.18 [95% CI, −0.02 to 0.37]; *P*=0.072). **E**, EGFr 1–6 patients have a significantly higher burden of enlarged perivascular spaces than EGFr 7–34 patients in the ATL (*P*=8.3×10^-7^) and subinsular regions (*P*=0.006), but not in the basal ganglia (*P*=0.10) or the white matter (*P*=0.49). The odds ratio is shown for EGFr 1–6 patients compared to EGFr 7–34 patients (reference group: odds ratio, 1).

**Figure 4.**
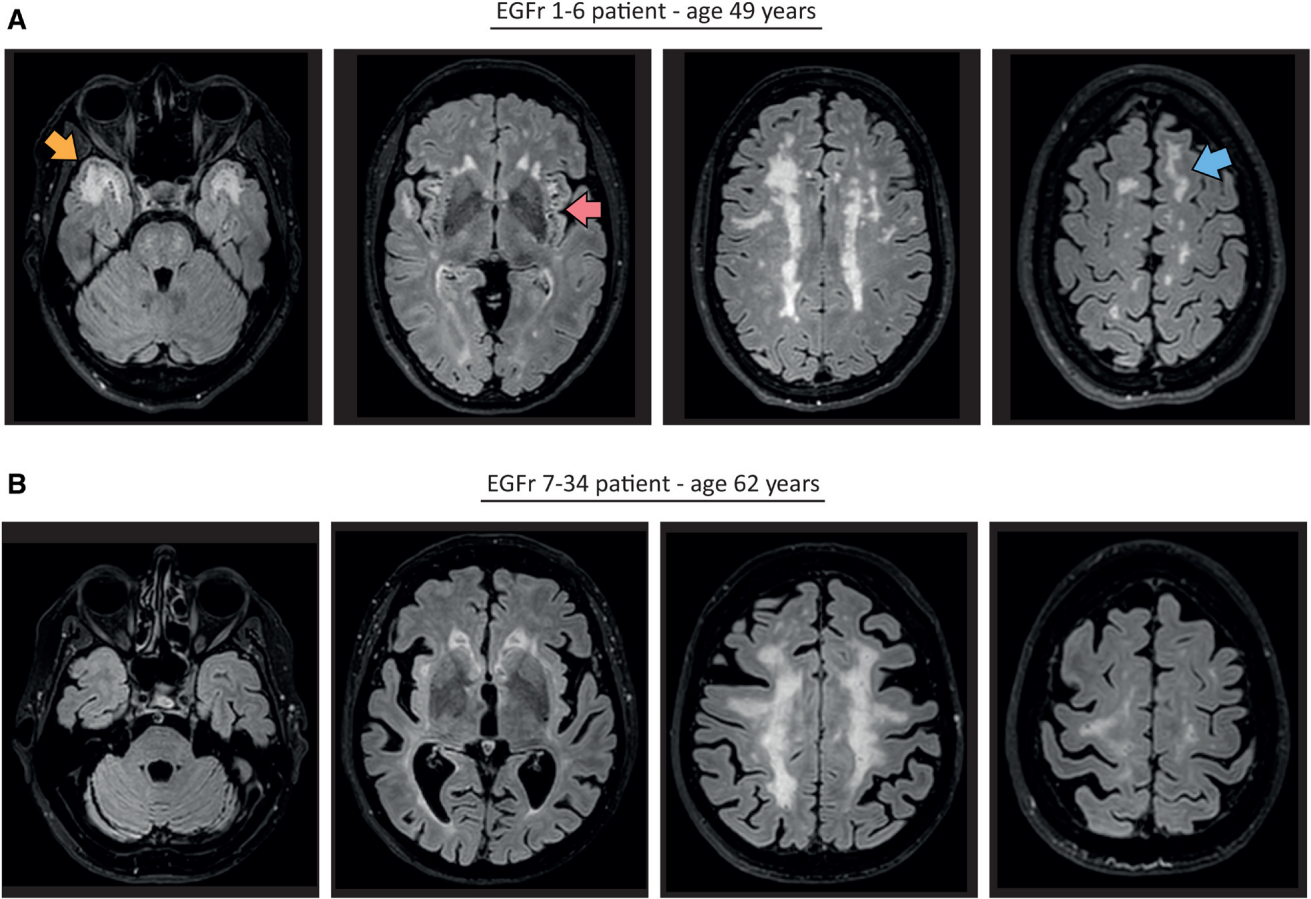
**Representative brain magnetic resonance imaging (MRI) of an EGFr (epidermal growth factor-like repeat) 1–6 and an EGFr 7–34 patient with cerebral autosomal dominant arteriopathy with subcortical infarcts and leukoencephalopathy (CADASIL). A**, Brain MRI FLAIR images of an EGFr 1–6 patient aged in his late 40s showing large confluent white matter hyperintensity (WMH) and a high burden of enlarged perivascular spaces (ePVS) in the anterior temporal lobes (yellow arrow), a high burden of ePVS in the subinsular regions (red arrow), and confluent WMH in the superior frontal gyri (blue arrow). **B**, Brain MRI fluid-attenuated inversion recovery images of an EGFr 7–34 patient aged in his early 60s with a similar total WMHv, but with no WMH in anterior temporal lobes, only small punctate foci of WMH in superior frontal gyri, and very low burden of ePVS in anterior temporal lobes and subinsular regions.

After adjustment for age, EGFr 1–6 patients had a significantly higher nWMHv (*P*=7.6×10^-13^), PSMD (*P*=2.2×10^-8^) and nLV (*P*=1.8×10^-5^; Figure 2 and Table S3). Additionally, EGFr 1–6 patients had a significantly higher nWMHv in the ATL and SFG (*P*=2.1×10^-15^), which was still significant after adjustment for total nWMHv (*P*=4.7×10^-4^; Figure 3A through 3D and Figure 4). EGFr 1–6 patients also had a significantly higher burden of ePVS than EGFr 7–34 patients in the ATL (*P*=8.3×10^-7^) and subinsular regions (*P*=0.006), but not in the basal ganglia or the white matter (Figure 3E). There were no significant differences in CMB count. EGFr 1–6 patients had a higher BPF than EGFr 7–34 patients (*P*=0.045), but this was no longer significant after adjustment for nWMHv in the ATL and SFG (Table S4).

In multivariable analysis, the following SVD imaging markers were significantly associated with mRS score: BPF (*P*=5.3×10^-6^), nLV (*P*=0.004) and unspecific nWMHv (*P*=0.009; Table S5). There was no independent association between mRS score and nWMHv in the ATL and SFG (*P*=0.46) or ePVS score in any region.

### Relative Contribution of EGFr Group, Sex, and Cardiovascular Risk Factor Burden on CADASIL Disease Severity

**Figure 5.**
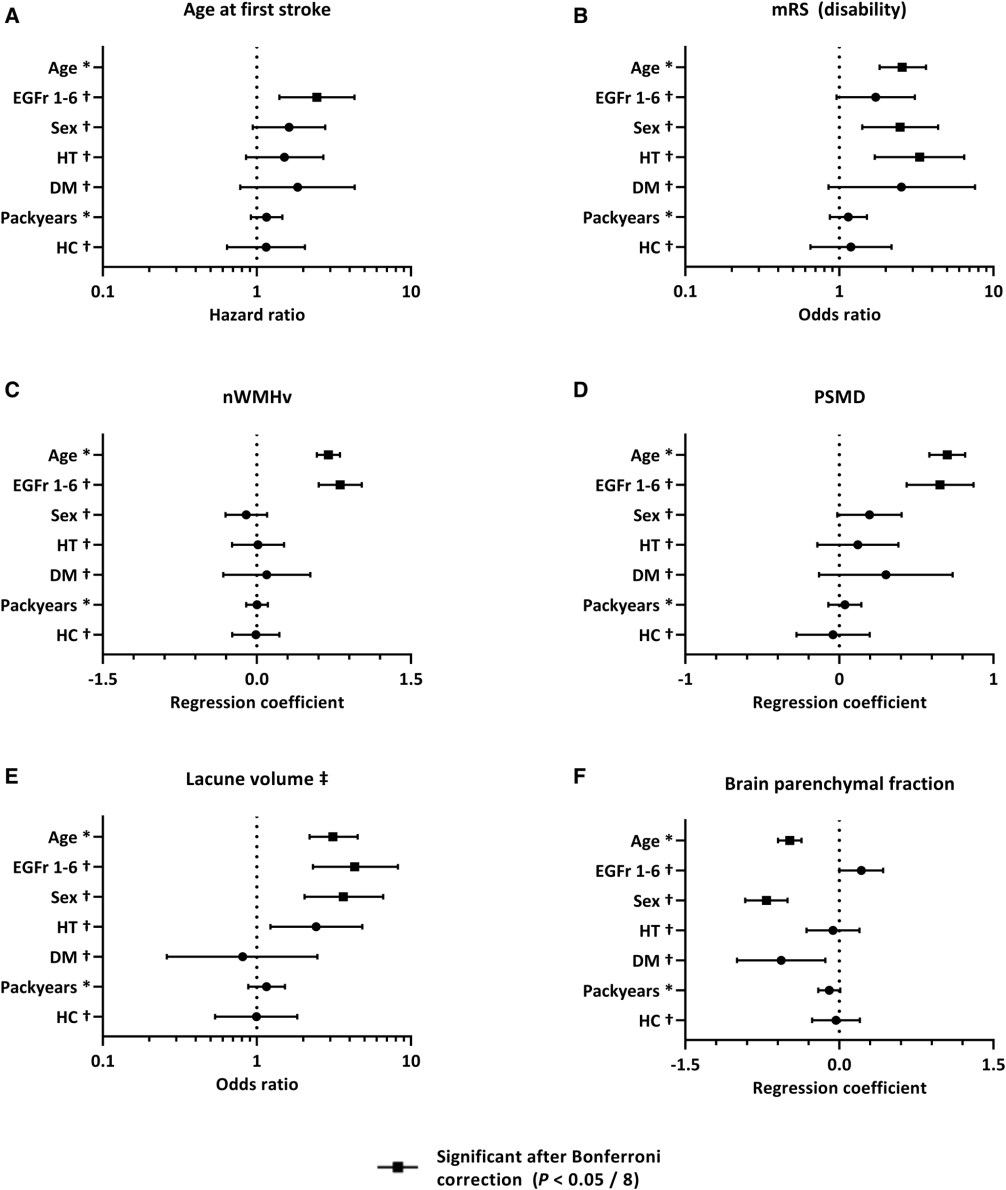
**Effect of cysteine altering missense *NOTCH3* variant (*NOTCH3*^cys^) EGFr (epidermal growth factor-like repeat) group, sex, and cardiovascular risk factors on cerebral autosomal dominant arteriopathy with subcortical infarcts and leukoencephalopathy (CADASIL) disease severity.** Forest plots showing the effect of EGFr group, sex and cardiovascular risk factors on clinical outcomes (**A** and **B**) and SVD imaging markers (**C–F**) in multivariable models expressed as hazard ratios, odds ratios and as regression coefficients. All dependent and independent continuous variables were standardized by dividing their value by their SD. DM indicates diabetes; HC, hypercholesterolemia; HT, hypertension; mRS, modified Rankin Scale; nWMHv, normalized white matter hyperintensity volume; and PSMD, peak width of the skeletonized mean diffusivity. *Hazard ratio (HR)/odds ratio (OR)/B is shown per SD of the variable. †HR/OR/B is shown for the presence of variable. ‡=normalized lacune volume was stratified in 4 categories (0; 0–0.005; 0.005–0.025; >0.025).

In multivariate analysis including EGFr group, sex, and CVRF, only EGFr 1–6 group was significantly associated with an earlier onset of stroke (hazard ratio, 2.45 [95% CI, 1.39–4.31]; *P*=0.002; Figure 5A). There was a trend towards an earlier onset of stroke in males (hazard ratio, 1.62 [95% CI, 0.94–2.78]; *P*=0.084). For disability, the most important modifier was hypertension (OR, 3.32 [95% CI, 1.70–6.47]; *P*=4.3×10^-4^), followed by male sex (OR, 2.48 [95% CI, 1.41–4.38]; *P*=0.002), diabetes (OR, 2.53 [95% CI, 0.85–7.59]; *P*=0.097) and EGFr 1–6 group (OR, 1.72 [95% CI, 0.96–3.09]; *P*=0.069; Figure 5B). The only disease modifier which was significantly associated with processing speed was packyears of smoking (B=−0.18 [95% CI, −0.32 to −0.03]; *P*=0.017; Figure S1A).

In multivariate analysis of SVD imaging markers, EGFr 1–6 group was the only disease modifier significantly associated with a higher nWMHv (B=0.81 [95% CI, 0.60–1.02]; *P*=1.1×10^-12^) and PSMD (B=0.65 [95% CI, 0.44–0.87]; *P*=1.6×10^-8^; Figure 5C and 5D). The most important modifier for nLV was EGFr 1–6 group (OR, 4.31 [95% CI, 2.31–8.04]; *P*=4.0×10^-6^), followed by male sex (OR, 3.64 [95% CI, 2.03–6.54]; *P*=1.5×10^-5^) and hypertension (OR, 2.43 [95% CI, 1.23–4.81]; *P*=0.011; Figure 5E); for BPF, the most important modifier was male sex (B=−0.71 [95% CI, −0.92 to −0.50]; *P*=1.4×10^-10^), followed by diabetes (B=−0.57 [95% CI, −1.00 to −0.14]; *P*=0.010), EGFr 1–6 group (B=0.21 [95% CI, 0.00–0.43]; *P*=0.050) and packyears (B=−0.10 [95% CI, −0.21 to 0.01]; *P*=0.073; Figure 5F); for CMB, this was hypertension (OR, 2.73 [95% CI, 1.36–5.46]; *P*=0.005), followed by male sex (OR, 2.05 [95% CI, 1.13–3.75]; *P*=0.019; Figure S1B). Nonfasting levels of LDL-C, HDL-C, triglycerides, hemoglobin A1C were not significantly associated with any of the clinical outcomes or neuroimaging markers in multivariable models.

## Discussion

In this prospective, genotype-driven, single-center CADASIL cohort study, addressing for the first time the relative disease-modifying effects of *NOTCH3*^cys^ EGFr group, CVRF, and sex on CADASIL disease severity, we show that EGFr group is the strongest disease modifier in CADASIL. EGFr 1–6 patients with CADASIL have an earlier onset of ischemic events, a higher burden of SVD neuroimaging markers, and a lower survival than EGFr 7–34 patients. The fact that EGFr 7–34 patients are milder is in line with the fact that EGFr 7–34 variants are frequent in the general population (1:300–1:1000), and in these population cohorts are associated with a very broad spectrum of cerebral SVD and even nonpenetrance.^3-5^ Therefore, EGFr 7–34 patients in CADASIL cohorts, although milder than EGFr 1–6 patients, still constitute the severe end of the EGFr 7–34 SVD spectrum.

A novel finding of this study is that *NOTCH3*^cys^ EGFr 1–6 variants are not only associated with a higher WMH volume and an earlier onset of stroke^5-7^ but also with other important CADASIL neuroimaging markers which are associated with disease severity, namely lacune volume and microstructural white matter damage as measured by DTI,^15,16,27^ as well as enlarged perivascular spaces. These findings can improve CADASIL patient stratification and inclusion for clinical studies, as the most severe patients are clearly those with EGFr 1–6 variants, and their disease severity is reflected by the clinically most relevant neuroimaging markers.^15,16,27^

EGFr 1–6 patients had a higher burden of WMH and ePVS in CADASIL-specific regions, that is, ATL, SFG and subinsular region, previously shown to be associated with increased water content and brain swelling.^25,26^ Indeed, we found that nWMHv in CADASIL-specific regions was significantly associated with a higher BPF. Possibly, *NOTCH*3^cys^-EGFr 1–6 variants lead to a more pronounced blood-brain barrier dysfunction in these regions.^29,30^

Despite the substantially higher burden of SVD neuroimaging markers, EGFr 1–6 patients did not have higher disability scores than EGFr 7–34 patients, and they performed equally well on cognitive tests. Potential differences in cognitive function and disability may have been masked by the fact that EGFr 1–6 patients had a higher educational level than the EGFr 7–34 participants, which has been shown to protect against the detrimental effects of brain damage in patients with CADASIL.^31^ In addition, older EGFr 1–6 patients were underrepresented because they were more likely to be too disabled to participate or to be deceased, or were diagnosed with CADASIL at a younger age.

EGFr 1–6 variants are extremely rare in the population, which has been the rationale for the cutoff between EGFr group 1–6 and EGFr group 7–34 in this and other studies.^5-7^ A recent study points towards a potential pathomechanistic explanation for the differences between EGFr groups, as EGFr 1–6 variants were shown to be associated with significantly higher NOTCH3 aggregation load in CADASIL vessels than EGFr 7–34 variants.^32^ However, there are some variants in EGFr 7–34 which are frequent in numerous CADASIL cohorts, suggesting that some EGFr 7–34 variants may be more severe and the current 1–6 versus 7–34 model may be too simplistic.

After EGFr group, we found sex to be the most important disease modifier; male sex was associated with increased disability, risk of stroke, lacune volume, CMB count, and lower BPF. This important modifying effect of sex has not been consistently observed in prior studies^7,9,11,14,16,33,34,6,13,12,15,13,10,13^. The fact that we were able to discern this effect is likely due to our study design, which includes not only EGFr group but also all CVRF. Possibly, the effect of sex is modulated by the enhanced vasodilatory and neuroprotective effect of premenopausal female hormones.^35^

Hypertension has been described to be associated with a number of neuroimaging markers and stroke in patients with CADASIL.^8-12,16^. Here, we found that hypertension was significantly associated with nLV, CMB, and disability but not with nWMHv^9^ and stroke.^12^ Also, packyears of smoking were associated with a lower brain volume and lower processing speed in DiViNAS participants but not with an increased risk of stroke as 2 prior studies.^12,15^ Differences in study design, cohort composition and definition of CVFR likely account for the differences that are found between CVFR and disease severity measures in the large CADASIL cohort studies performed to date.^6,7,12,13,15,16^ Taken together, however, hypertension is consistently found to be an important and actionable disease modifier in CADASIL, which suggests blood pressure monitoring and antihypertensive treatment may be beneficial for patients with CADASIL. However, therapeutic trials are warranted as cerebrovascular reactivity and autoregulation are impaired and lowering blood pressure could cause cerebral hypoperfusion.^36^ In contrast, in line with the European Academy of Neurology consensus statement,^37^ standard preventive prescription of statins seems not to be indicated in patients with CADASIL with normal lipid levels, as hypercholesterolemia has never been found to be associated with any of the CADASIL outcome measures, a finding which we corroborate in this study.^6,7,9-12,14-17,33,34^ However, statin therapy is not contraindicated in patients with CADASIL, and statins can be prescribed for other indications such as coronary artery disease.

Only 13 individuals in the DiViNAS cohort had diabetes type I or II, but despite the small sample size we still found a significant association between diabetes and a lower BPF. There was also a trend towards higher disability in DiViNAS participants with diabetes, suggesting a potential strong effect of diabetes on CADASIL disease expression. Larger cohorts are needed to accurately estimate the effect of diabetes on CADASIL disease severity.

Although EGFr group and CVRF have now unequivocally been shown to be important CADASIL disease modifiers, a significant proportion of disease variability is still unaccounted for. Identifying these (genetic) modifiers will contribute to improved personalized disease prediction and may lead to the identification of novel biological treatment targets for CADASIL.

A limitation of this study is the selection bias towards relatively mildly affected patients, and the consequent relatively low frequency of clinical stroke in this cohort. Strengths are the genotype-driven study design, the prospectively and uniformly collected data including high-resolution neuroimaging, and the fact that both EGFr groups are equally represented.

In conclusion, this prospective, genotype-driven cohort study has validated and extended the substantial differences in disease severity between EGFr 1–6 and 7–34 CADASIL patient. Our results show that *NOTCH3*^cys^ EGFr group is the most important modifier of CADASIL known to date, followed by male sex, hypertension, diabetes, and smoking. These findings are important for the improvement of disease prediction, for guiding patient inclusion in clinical trials and for developing guidelines for secondary disease prevention.

## Article Information

### Acknowledgments

The authors wish to acknowledge all the patients with cerebral autosomal dominant arteriopathy with subcortical infarcts and leukoencephalopathy (CADASIL) who participated in our studies. Dr Hack designed and conceptualized the study and contributed to acquisition of data, analyzed and interpreted the data, and drafted the manuscript. Dr Cerfontaine and S. Tap contributed to acquisition of data and critical revision of the manuscript for important intellectual content. Drs Gravesteijn, Hafkemeijer, van der Grond, Witjes-Ané, and Baas contributed to critical revision of the manuscript for important intellectual content. Dr Rutten and Lesnik Oberstein designed and conceptualized the study and contributed to acquisition of data, interpreted the data, critical revision of the manuscript for important intellectual content, and supervision.

### Sources of Funding

This study was funded by Netherlands Organisation for Health Research and Development (ZonMW 91717325) and The Netherlands Brain Foundation (HA2016-02-03).

### Disclosures

Dr Hack and Cerfontaine are funded by the Netherlands Organisation for Health Research and Development (ZonMW 91717325). Dr Rutten has financial research support from the Netherlands Organisation for Health Research and Development (ZonMW 91717325) and the Netherlands Brain Foundation (HA2016-02-03). Dr Lesnik Oberstein has financial research support from the Netherlands Organisation for Health Research and Development (ZonMW 91717325) and the Netherlands Brain Foundation (HA2016-02-03), and receives grants from Leiden University Medical Center. The other authors report no conflicts.

### Supplemental Material

Supplemental Methods

Table S1–S5

Figure S1

STROBE Checklist

Reference 18,20,21,23,25-27

## Supplementary Material


