# Supplementary material for: Effect of NOTCH3 EGFr Group, Sex, and Cardiovascular Risk Factors on CADASIL Clinical and Neuroimaging Outcomes
Source: Stroke. 2022 Jul 13;53(10):3133–44. doi: 10.1161/STROKEAHA.122.039325 (PMC9508953; doi:10.1161/STROKEAHA.122.039325)
Supplement: Supplementary file 1 [file str-53-3133-s001.pdf]

SUPPLEMENTAL MATERIAL

The effect of *NOTCH3* EGFr group, sex and cardiovascular risk factors on CADASIL clinical and neuroimaging outcomes.

Remco J. Hack, MD[1]; Minne N. Cerfontaine, MD[1]; Gido Gravesteijn, MD, PhD[1]; Stephan Tap, MSc[1]; Anne Hafkemeijer, PhD[2, 3, 4]; Jeroen van der Grond, PhD[2]; Marie-Noëlle Witjes-Ané, MD, PhD[5]; Frank Baas, MD, PhD[1]; Julie W. Rutten, MD, PhD[1] †; Saskia A.J. Lesnik Oberstein, MD, PhD[1] †.

[1] Department of Clinical Genetics, Leiden University Medical Center, Leiden, the Netherlands.

[2] Department of Radiology, Leiden University Medical Center, Leiden, the Netherlands.

[3] Institute of Psychology, Leiden University, Leiden, the Netherlands.

[4] Leiden Institute for Brain and Cognition, Leiden University, Leiden, the Netherlands.

[5] Department of Geriatrics and Psychiatrics, Leiden University Medical Center, Leiden, the Netherlands.

† shared last authors

Supplemental Methods

Assessment of clinical symptoms and cardiovascular risk factors

Stroke and TIA were defined as rapidly evolving focal symptoms lasting >24 and <24 hours, respectively, with no apparent cause other than of vascular origin. Neuroimaging proven large vessel ischemic strokes (n=3) and cerebral venous thrombosis (n=1) were scored separately. Dementia was defined as a previous diagnosis of dementia or according to the Diagnostic and Statistical Manual of Mental Disorders, 5th edition (DSM-V). To assess if patients currently had apathy or other neuropsychiatric symptoms, relatives were interviewed in the clinic or by telephone using the Dutch version of the Neuropsychiatric Inventory (NPI). Apathy was defined as a previous diagnosis, or a NPI score of apathy ≥1 with a score on the Starkstein Apathy Scale of ≥14. Depression was defined as a previous diagnosis, or a NPI score of depression ≥1 with symptoms fulfilling the diagnostic criteria of DSM-V. Walking disability was defined as any gait impairment objectified during neurological examination at the study site. Migraine with and without aura were defined according the International Classification of Headache Disorders 3rd edition. CADASIL encephalopathy was defined as an acute reversible encephalopathy with evidence of reduced consciousness lasting > 24 hours in absence of an organic cause which was sufficient to warrant hospital admission.[18] Disability was assessed using mRS. Affected parent's age at first stroke, current age or age at death was recorded.

Hypertension was defined as a previous diagnosis of hypertension (>140 mmHg systolic or >90 mmHg diastolic) or use of an antihypertensive agent. Hypercholesterolemia was defined as a previous diagnosis of hypercholesterolemia or non-fasting level of LDL-C > 3.5 mmol/l or total cholesterol > 6.5 mmol/l. Diabetes was defined as a previous diagnosis of diabetes type 1 or 2 or use of a hypoglycemic agent.

Calculation of cognitive test scores

Performances in each cognitive test (except MOCA) were converted to t-scores using normative data reported in the literature.[20] The age, sex and educational level according to Verhage of each participant were considered when converting their raw scores to t-scores, except for t-scores of WAIS cognitive tests (Coding, Digit Span and Block Design), which only considered age.

For 14 participants only WAIS Coding, Digit Span and TMTA&B were available as these individuals were participants of the P17.170 study,[21] which had a different neuropsychological test battery. Exclusion of participants from the P17.170 study did not change the results of analyses with cognitive scores as outcomes.

Acquisition parameters of brain MRI

The following sequences were obtained: three dimensional T1-weighted images (echo time [TE] 3.5 ms, repetition time [TR] 7.9 ms, Flip 8°, voxel size 1.04 x 1.04 x 1.10 mm, matrix size 240×240×155 mm), T2-weighted images (TE/TR/Flip: 80ms/3.0s/90°, voxel size 0.40x0.40x5.0 mm, matrix size 576×576×30), fluid-attenuated inversion recovery (TE/TR/Flip angle: 250ms/4.8s/90°, voxel size 0.74x0.74x0.56 mm, matrix size 336x336x326) and susceptibility-weighted images (TE/TR/Flip angle: 31ms/45ms/17°, voxel size: 0.40×0.40×1.0 mm, matrix size 576x576x145). Echoplanar imaging was used to obtain single-shell diffusion-weighted imaging sequences: repetition time 6250 ms, echo time 72 ms, diffusion encoding direction 30 (b=1000 s/mm^2), voxel size 2.0 x 2.0 x 2.1 mm, FOV 224x224x126 mm. One b=0 with inverted phase encoding direction was obtained to correct for susceptibility distortions.

Quantification of SVD imaging markers

WMHv were calculated using a WMH mask generated from FLAIR images, including hyperintensities in subcortical gray matter structures and brain stem, using Brain Intensity AbNormality Classification Algorithm (BIANCA) with settings optimized for CADASIL populations.[27] Thresholds for WMH masks

were individually selected and manually corrected if necessary. Number and volume of lacunes were evaluated on T1-weighted images. CMB were evaluated on SWI images according the Microbleed Anatomical Rating Scale (MARS). T1-weighted images were used to assess brain parenchymal volume with SIENAX and estimated total intracranial volume (eTIV) was assessed with Freesurfer v.7.1.1 3 (http://surfer.nmr.mgh.harvard.edu). ePVS were evaluated in four specific regions: the global white matter, basal ganglia, subinsular region and anterior temporal poles (ATL), according to a 4-grade semiquantitative scale.[23] ePVS were counted bilaterally on the slice with the most ePVS for each specific region on T1-weighted images in association with T2-weighted and FLAIR images. FMRIB Software Library (FSL) v6.0.4, (Analysis Group, FMRIB, Oxford, UK) was used for volumetric analyses of lacunes and WMH.

Twenty randomly selected brain MRIs were evaluated by a second observer for lacunes, CMB count and ePVS score to assess interrater reliability. A high interrater reliability was demonstrated between for lacune count (intraclass correlation coefficient (ICC) = 0.94, 95% CI 0.86 – 0.97), lacune volume (ICC = 0.97, 95% CI 0.93 – 0.99), cerebral microbleed count (ICC = 0.92, 95% CI 0.82 – 0.97) and total ePVS count (weighted kappa with linear weights = 0.72, 95% CI 0.62 – 0.83). Weighted kappa and ICC and their 95% confident intervals were calculated using SPSS v27.0. ICC calculations were based on single measurements, absolute-agreement and 2-way random effects.

Quantification of WMH in anterior temporal lobes and superior frontal gyri

To define the region of interest (ROI) for the ATL and SFG, we used the structural labels of the digitized version of the Talairach atlas registered into the Montreal Neurological Institute (MNI) template.[25] To register the ROI mask to the FLAIR images, FLAIR images were first registered to the MNI-152 template using FLIRT (https://fsl.fmrib.ox.ac.uk/fsl/fslwiki/FLIRT) and FNIRT (https://fsl.fmrib.ox.ac.uk/fsl/fslwiki/FNIRT) registration algorithms of FSL, after which the inverted warp was used to register the ROI mask to the FLAIR image. The ROI mask was used to calculate the WMHv in ATL and SFG.

DTI pre-processing and metrics

The following preprocessing steps were performed: visual quality control, denoising, Gibbs artefact removal and correction for susceptibility distortion, eddy currents, head motion and bias field. This was done using software of MRtrix3 v3.0.3 (*dwidenoise, mrdegibbs* and *dwibiascorrect with Advanced Normalization Tools*) and Functional Magnetic Resonance Imaging of the Brain Software Library (FSL) v6.0.4 (*topup* and *eddy*).

To obtain diffusion metrics within the major white matter tracts, diffusion data was skeletonized using the tract based spatial statistics (TBSS) pipeline in FSL. The skeleton mask was obtained by: 1) registering participants' FA maps to the standard space FMRIB 1mm FA template using the nonlinear registration tool FNIRT, 2) thresholding the mean FA map at an FA value of 0.2 and 3) manually removing areas susceptible for CSF partial volume effects from the skeleton mask. Mean diffusivity (MD), axial diffusivity (AD) and radial diffusivity (RD) images were projected onto the skeleton using FA-derived projection parameters. The average values of MD and FA within the entire skeleton were used in all analyses.

Peak width of the Skeletonized Mean Diffusivity (PSMD), a proven robust imaging marker for SVD,[26] which is the difference between the 95th and 5th percentiles of the voxel-based MD values within the skeleton, was calculated by using a fully-automated shell script (http://www.psmd-marker.com/) on our pre-processed diffusion weighted images.[26] *DTIFIT* in FSL was used to calculate the following diffusion metrics: fractional anisotropy, mean-, axial- and radial diffusivity.

Supplemental Tables

Table S1. *NOTCH3*^{cys} variants in the DiViNAS cohort.

Nucleotide alteration	Protein alteration	Exon	EGFr domain	Count	Percentage
c.160C>T	p.Arg54Cys	2	1	3	1,5
c.328C>T	p.Arg110Cys	3	2	5	2.5
c.350G>A	p.Cys117Tyr	4	2	2	1.0
c.397C>T	p.Arg133Cys	4	3	3	1.5
c.421C>T	p.Arg141Cys	4	3	16	8.0
c.431G>T	p.Cys144Phe	4	3	4	2.0
c.457C>T	p.Arg153Cys	4	3	11	5.5
c.486C>G	p.Cys162Trp	4	4	3	1.5
c.505C>T	p.Arg169Cys	4	4	3	1.5
c.544C>T	p.Arg182Cys	4	4	13	6.5
c.548G>A	p.Cys183Tyr	4	4	2	1.0
c.619C>T	p.Arg207Cys	4	5	28	14.0
c.634T>A	p.Cys212Ser	4	5	1	0.5
c.665G>A	p.Cys222Tyr	4	5	1	0.5
c.671G>A	p.Cys224Tyr	4	5	1	0.5
c.698G>A	p.Cys233Tyr	5	5	1	0.5
c.953G>T	p.Cys318Phe	6	8	2	1.0
c.1130G>A	p.Cys377Tyr	7	9	1	0.5
c.1187C>G	p.Ser396Cys	7	10	2	1.0
c.1261C>T	p.Arg421Cys	8	10	1	0.5
c.1279C>T	p.Arg427Cys	8	10	1	0.5
c.1345C>T	p.Arg449Cys	8	11	2	1.0
c.1591T>G	p.Cys531Gly	10	13	2	1.0
c.1630C>T	p.Arg544Cys	11	14	2	1.0
c.1672C>T	p.Arg558Cys	11	14	1	0.5
c.1703G>A	p.Cys568Tyr	11	14	5	2.5
c.1732C>T	p.Arg578Cys	11	14	45	22.5
c.1759C>T	p.Arg587Cys	11	15	2	1.0
c.1783G>T	p.Gly595Cys	11	15	2	1.0
c.1819C>T	p.Arg607Cys	11	15	3	1.5
c.1999G>T	p.Gly667Cys	13	17	9	4.5
c.2182C>T	p.Arg728Cys	14	18	3	1,5
c.3043T>C	p.Cys1015Arg	19	26	12	6.0
c.3091C>T	p.Arg1031Cys	19	26	2	1.0
c.3226C>T	p.Arg1076Cys	20	27	4	2.0
c.3691C>T	p.Arg1231Cys	22	31	2	1.0
**Total**				**200**	**100**

Table S2. Cognitive test scores of DiViNAS cohort.

	NOTCH3[cys] EGFr 1-6	*NOTCH3*[cys] EGFr 7-34	P_{age}*
**Raw scores**			
MOCA, median (IQR)	26 (4)	24 (4)	0.86 †
**T-scores ‡**			
TMTA, mean (SD)	49.9 (15.1)	49.8 (14.5)	0.71
TMTB, mean (SD)	46.6 (14.4)	45.5 (14.5)	0.90
Stroop IF, mean (SD)	52.4 (8.7)	52.7 (9.0)	0.44
RAVLT learning, mean (SD)	34.9 (10.5)	36.5 (12.0)	0.18
RAVLT recall, mean (SD)	39.1 (12.6)	39.8 (12.7)	0.17
VF animal, mean (SD)	46.0 (11.9)	46.8 (10.6)	0.38
VF professions, mean (SD)	45.5 (12.7)	44.5 (10.5)	0.99
Coding, mean (SD)	51.4 (9.1)	49.3 (9.1)	0.37
Digit Span, mean (SD)	47.9 (9.8)	45.5 (10.6)	0.16
Block Design, mean (SD)	47.8 (11.1)	47.6 (11.6)	0.46

Abbreviations: TMTA/B = Trail Making Test A/B; RAVLT = Rey Auditory Verbal Learning Test; Stroop IF = Stroop Interference; VF = Verbal Fluency

* = *P*-value corrected for age

† = *P*-value corrected for age and educational level

‡ = T-scores were calculated based on normative data based on age, sex and/or educational level depending on the cognitive test

Table S3. SVD neuroimaging lesion load of DiViNAS cohort.

		*NOTCH3^cys^* EGFr 1-6	*NOTCH3^cys^* EGFr 7-34	P_{age}*
**nWMHv, mean (SD)**	**ATL**	0.21 (0.26)	0.08 (0.14)	**$5.8x10^{-15}$**
	**SFG**	0.15 (0.21)	0.06 (0.10)	**$1.6x10^{-10}$**
	**total**	4.0 (2.9)	2.8 (2.4)	**$7.6x10^{-13}$**
**nLV, median (IQR)**	**total ($x10^{-3}$)**	3.01 (25.83)	0.29 (9.14)	**$1.8x10^{-5}$**
**BPF, mean (SD)**		71.1 (4.0)	69.1 (3.7)	**0.045**
**CMB count, median (IQR)**	**deep**	0 (2)	0 (2)	0.11
	**lobar**	0 (0)	0 (2)	0.84
	**infratentorial**	0 (0)	0 (0)	0.13
	**total**	0 (4)	0 (6)	0.21
**ePVS score, median (IQR)**	**white matter**	2 (2)	2 (2)	0.49
	**basal ganglia**	2 (1)	2(1)	0.10
	**subinsular**	2 (1)	2 (1)	**0.006**
	**ATL**	2 (2)	1 (1)	**$8.3x10^{-7}$**
	**total**	7 (5)	7 (4)	**0.001**
**DTI, median (IQR)**	**MD ($x10^4$)**	8.29 (1.10)	8.07 (1.13)	**$2.6x10^{-10}$**
	**PSMD ($x10^4$)**	3.58 (2.30)	3.35 (2.21)	**$2.2x10^{-8}$**
	**FA**	0.42 (0.07)	0.43 (0.06)	**$1.3x10^{-8}$**
	**AD ($x10^4$)**	12.42 (0.79)	12.25 (0.86)	**$6.2x10^{-11}$**
	**RD ($x10^4$)**	6.22 (1.27)	6.01 (1.21)	**$6.1x10^{-10}$**

Abbreviations: AD = axial diffusivity; ATL = anterior temporal lobe; BPF = brain parenchymal fraction; CMB = cerebral microbleed; DTI = diffusion tensor imaging; ePVS = enlarged perivascular spaces; FA = fractional anisotropy; MD = mean diffusivity; PSMD = peak width of skeletonized mean diffusivity; nLV = normalized lacune volume; nWMHv = normalized white matter hyperintensity volume; RD = radial diffusivity; SFG = superior frontal gyrus.

* = *P*-value corrected for age

Table S4. Association between brain parenchymal fraction and other neuroimaging small vessel disease markers.

	Model 1 *		Model 2 *		Model 3 *	
	**B (95% CI)**	***P***	**B (95% CI)**	***P***	**B (95% CI)**	***P***
**Age †**	-0.56 (-0.71 – -0.41)	**$3.8x10^{-12}$**	-0.51 (-0.65 – -0.35)	**$4.0x10^{-10}$**	-0.50 (-0.65 – -0.36)	**$2.2x10^{-10}$**
**nLV †**	-0.34 (-0.49 – -0.19)	**$1.1x10^{-5}$**	-0.35 (-0.49 – -0.20)	**$5.3x10^{-6}$**	-0.35 (-0.49 – -0.20)	**$5.0x10^{-6}$**
**Total CMB count †**	0.02 (-0.12 – 0.16)	0.77	0.06 (-0.08 – 0.21)	0.37	0.06 (-0.08 – 0.20)	0.39
**ePVS white matter ‡**	0.08 (-0.18 – 0.34)	0.55	0.08 (-0.17 – 0.34)	0.52	0.08 (-0.18 – 0.34)	0.54
**ePVS basal ganglia ‡**	-0.29 (-0.58 – 0.00)	0.052	-0.16 (-0.45 – 0.14)	0.29	-0.16 (-0.45 – 0.13)	0.28
**ePVS subinsular ‡**	-0.25 (-0.61 – 0.11)	0.17	-0.28 (-0.63 – 0.08)	0.12	-0.27 (-0.62 – 0.08)	0.13
**ePVS ATL ‡**	0.57 (0.23 – 0.90)	**$9.9x10^{-4}$**	0.37 (0.01 – 0.72)	**0.041**	0.36 (0.01 – 0.71)	**0.044**
**Total nWMHv †**	0.25 (0.09 – 0.42)	**0.003**	-	**-**	-	-
**ATL nWMHv †**	-	-	0.27 (0.03 – 0.51)	**0.029**	-	-
**SFG nWMHv †**	-	-	0.13 (-0.11 – 0.37)	0.29	-	-
**ATL + SFG nWMHv †**	-	-	-	**-**	0.39 (0.18 – 0.60)	**$4.1x10^{-4}$**
**Unspecific nWMHv † ‡**	-	-	-0.12 (-0.37 – 0.14)	0.36	-0.12 (-0.37 – 0.14)	0.37

Abbreviations: ATL = anterior temporal lobes; CMB = cerebral microbleed; nLV = normalized lacune volume; nWMHv = normalized white matter hyperintensity volume; SFG = superior frontal gyrus.

* = multivariable model.

† = continuous variables were standardized by dividing their value by their standard deviation.

‡ = ePVS scores per region were stratified in two categories (score 1-2 and 3-4).

§ = unspecific nWMHv is defined as the total WMHv subtracted by the WMHv in the ATL and SFG.

Table S5. Association between SVD imaging markers and disability.

	Model 1 *		Model 2 *		Model 3 *	
	OR (95% CI)	*P*	OR (95% CI)	*P*	OR (95% CI)	*P*
**Age †**	0.81 (0.50 – 1.34)	0.42	0.88 (0.53 – 1.46)	0.61	0.88 (0.54 – 1.43)	0.59
**nLV †**	1.86 (1.22 – 2.87)	**0.004**	1.91 (1.24 – 2.97)	**0.004**	1.68 (1.08 – 2.62)	**0.021**
**BPF †**	0.38 (0.25 – 0.57)	**5.3×10^{-6}**	0.37 (0.24 – 0.56)	**4.8×10^{-6}**	0.47 (0.31 – 0.71)	**3.7×10^{-4}**
**CMB count †**	1.05 (0.73 – 1.52)	0.78	0.99 (0.69 – 1.43)	0.97	1.01 (0.69 – 1.47)	0.97
**ePVS white matter ‡**	0.73 (0.34 – 1.54)	0.41	0.64 (0.29 – 1.38)	0.26	0.68 (0.31 – 1.46)	0.33
**ePVS basal ganglia ‡**	1.71 (0.80 – 3.69)	0.17	1.76 (0.81 – 3.85)	0.15	1.94 (0.89 – 4.26)	0.10
**ePVS subinsular ‡**	1.10 (0.45 – 2.72)	0.83	1.09 (0.44 – 2.72)	0.85	0.84 (0.31 – 2.21)	0.72
**ePVS ATL ‡**	1.23 (0.51 – 2.93)	0.65	1.30 (0.53 – 3.14)	0.56	1.28 (0.51 – 3.19)	0.60
**Unspecific nWMHv † §**	2.72 (1.29 – 5.83)	**0.009**	2.81 (1.31 – 6.09)	**0.008**	-	-
**nWMHv in ATL & SFG †**	0.77 (0.38 – 1.56)	0.46	-	-	-	-
**nWMHv ATL †**	-	-	0.99 (0.45 – 2.15)	0.96	-	-
**nWMHv SFG †**	-	-	0.74 (0.39 – 1.44)	0.36	-	-
**PSMD †**	-	-	-	-	2.43 (1.36 – 4.43)	**0.003**

Abbreviations: ATL = anterior temporal lobes; BPF = brain parenchymal fraction; CMB = cerebral microbleed; ePVS = enlarged perivascular spaces; nLV = normalized lacune volume; nWMHv = normalized white matter hyperintensity volume; PSMD = Peak width of Skeletonized Mean Diffusivity; SFG = superior frontal gyrus.

***** = multivariable model.
† = continuous variables were standardized by dividing their value by their standard deviation.
‡ = ePVS scores per region were stratified in two categories (score 1-2 and 3-4).
§ = unspecific nWMHv is defined as the total WMHv subtracted by the WMHv in the ATL and SFG.

Supplemental Figures

Figure S1

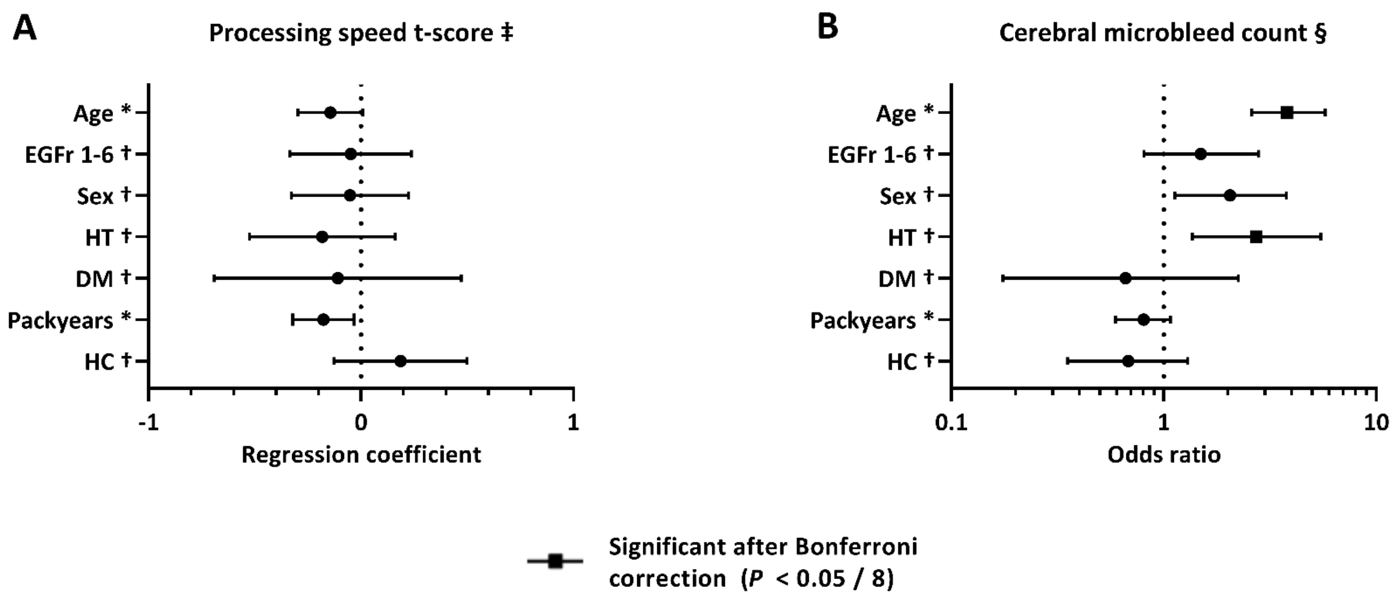

Figure S1. Effect of *NOTCH3*^{cys} EGFr group, sex and cardiovascular risk factors on CADASIL disease severity.

Forest plots showing the effect of EGFr group, sex and cardiovascular risk factors on processing speed (**A**) and cerebral microbleed count (**B**) in multivariable models expressed as regression coefficients and odds ratios. All dependent and independent continuous variables were standardized by dividing their value by their standard deviation.

Abbreviations: DM = diabetes mellitus; HC = hypercholesterolemia; HT = hypertension.

* = HR/OR/B is shown per standard deviation of the variable.
† = HR/OR/B is shown for the presence of variable.
‡ = compound z-score of Trail Making Test A and B
§ = total CMB was stratified in four categories (0; 1-4; 5-10; >10)
